# The Added Value of Vessel Wall MRI in the Detection of Intraluminal Thrombus in Patients Suspected of Craniocervical Artery Dissection

**DOI:** 10.14336/AD.2021.0502

**Published:** 2021-12-01

**Authors:** Yuehong Liu, Sijie Li, Ye Wu, Fang Wu, Ying Chang, Haibin Li, Xiuqin Jia, Luca Saba, Xunming Ji, Qi Yang, on behalf of the WISP Investigators

**Affiliations:** ^1^Department of Radiology, Beijing Chaoyang Hospital, Capital Medical University, Beijing, China.; ^2^Department of Radiology, Xuanwu Hospital, Capital Medical University, Beijing, China.; ^3^Department of Neurosurgery, Xuanwu Hospital, Capital Medical University, Beijing, China.; ^4^Department of Ultrasonography, Xuanwu Hospital, Capital Medical University, Beijing, China.; ^5^Department of Epidemiology, Beijing Chaoyang Hospital, Capital Medical University, Beijing, China.; ^6^Department of Radiology, Azienda Ospedaliero Universitaria (A.O.U.), Polo di Monserrato SS 554, Monserrato, Cagliari, Italy.; ^7^Beijing Laboratory for Cardiovascular Precision Medicine, Beijing, China.; ^8^Key Laboratory of Medical Engineering for Cardiovascular Disease, Ministry of Education, Beijing, China

**Keywords:** Craniocervical Artery Dissection, Vessel Wall MRI, DSA, Thrombus

## Abstract

Patients with craniocervical artery dissection (CCAD) have a high short-term risk of ischemic stroke, which is frequently associated with thromboembolism. Previous studies have demonstrated the utility of three-dimensional vessel wall MR imaging (3D-VWMRI) in the diagnosis of dissection. Few have investigated the value of 3D-VWMRI in the detection of intraluminal thrombus. The purpose of the current study was to evaluate the added value of 3D-VWMRI for thrombus identification in patients suspected of CCAD. One hundred and four patients (mean age, 44.2 years ± 13.2) suspected of CCAD and scheduled for digital subtraction angiography (DSA) were prospectively enrolled in the study and underwent VWMRI examination. The diagnostic performance of 3D-VWMRI for CCAD was evaluated using receiver operating characteristic (ROC) analysis with the final diagnosis results as the reference. The presence/absence of intraluminal thrombus on 3D-VWMRI/DSA was independently determined. The sensitivity and specificity of 3D-VWMRI for intraluminal thrombus detection were assessed with DSA serving as the reference. The odds ratio (OR) was used to evaluate the correlation between thrombus presented on 3D-VWMRI/DSA and ischemic stroke. The 3D-VWMRI had high sensitivity (90.0%) and specificity (94.3%) in identifying arteries with CCAD. The area under the ROC curve was 0.96. With DSA as the reference, the sensitivity and accuracy of 3D-VWMRI for the detection of intraluminal thrombus were 97.4% and 79.0%, respectively. An intraluminal thrombus present on 3D-VWMRI was strongly associated with a territorial ischemic stroke (OR: 30.0; 95% confidence interval: 9.1-98.4; *P* < .001). In conclusion, 3D-VWMRI with a 3.0-T MR system had a high diagnostic performance for CCAD and offered added value for detecting intraluminal thrombus.

Craniocervical artery dissection (CCAD) is a significant cause of stroke in young and middle-aged individuals [1, 2]. Patients with CCAD have a high short-term risk of ischemic stroke, and embolism from thrombus is the most frequent ischemic stroke mechanism after dissection [3]. Detection of intraluminal thrombus can facilitate the investigation of formation mechanisms and/or predict the risk for ischemic stroke in patients with suspected or predetermined CCAD. Clinicians routinely use antithrombotic or anticoagulant therapy to reduce the risk of worse outcomes caused by thrombosis for patients with cervical artery dissection [4]. Early determination of the CCAD diagnosis and the presence or absence of intraluminal thrombus is therefore of utmost importance to start timely and appropriate treatment. Conventional imaging methods, including MR angiography (MRA) and CT angiography (CTA), usually evaluate the presence of an intraluminal thrombus according to the occlusion of blood flow [5].

Vessel wall MRI (VWMRI) with blood flow and fat suppression greatly improves spatial resolution and is thus ideally suited to directly visualize the plaque, intramural hematoma (IMH), intimal flaps, double lumen, and thrombus [6, 7]. Recent studies suggested the clinical utility of VWMRI for investigating the etiology of acute ischemic stroke [8, 9]. A study demonstrated that VWMRI with three-dimensional T1 sampling perfection with application-optimized contrast using different flip angle evolution (3D T1-SPACE) sequences [10] offered excellent diagnostic value for cervical artery dissection on a 1.5-T MR system [11]. However, the efficacy of 3D T1-SPACE sequences for the detection of intraluminal thrombus in patients suspected of CCAD has not been previously reported. For patients with ischemic stroke but without occluded vessels, a modality for direct imaging of the intraluminal thrombus is required to further investigate the mechanisms and occurrence risk of ischemic stroke.

Here, we evaluated the diagnostic performance of 3D T1-SPACE sequences for CCAD using a 3.0-T MR system. Then, we sought to investigate the added value of 3D T1-SPACE sequences for intraluminal thrombus detection and to evaluate the association of nonocclusive thrombus detected using 3D T1-SPACE images for ischemic stroke in patients suspected of CCAD.

## MATERIALS AND METHODS

### Participants

Our study sample was based on the prospective population of the Whole Brain Vessel Wall Imaging in Stroke Patients (WISP) study. Local ethics committee approval and written informed patient consent were obtained for the current study. From March 2015 to July 2019, consecutive patients suspected of CCAD and scheduled for digital subtraction angiography (DSA) were prospectively invited for the 3D-VWMRI examination. Symptomatic patients or patients with acute cerebrovascular events were suspected of dissection if they had any suspicious pathognomonic signs of dissection (irregular stenosis, suspicious double lumen or intimal flap, and pseudoaneurysm) that presented on traditional imaging methods, including CTA, MRA, and ultrasonography. The exclusion criteria were as follows: a) missing data with ultrasonography and imaging data, including MRI of the brain, CTA, and/or MRA, b) evidence of cardioembolism, c) symptom onset time over 30 days, and d) inability to tolerate MRI and contrast-enhanced MRI (creatinine clearance < 60 mL/[min·1.73 m^2^]) or poor image quality for 3D-VWMRI considered inadequate for analysis. Demographic and clinical data, including age, sex, vascular risk factors, and the time interval between symptom onset and 3D-VWMRI, were recorded for each patient. The time interval between 3D-VWMRI and DSA was recorded for patients who had both 3D-VWMRI and DSA exams.

### VWMRI Protocol

All 3D-VWMRI images were obtained using a 3.0-T MRI scanner (Tim Trio or Verio, Siemens Healthcare, Erlangen, Germany) with a 32-channel head/neck coil and pre- and post-contrast sequences known as T1-SPACE in a sagittal orientation. Post-contrast vessel wall enhancement was used to characterize the vessel wall optimally and better identify the dissected lesions and intraluminal thrombus [12, 13]. The whole-brain VWMRI protocol recently proposed was adapted [14, 15]: repetition time = 900 ms; echo time = 14 ms; field of view = 230 mm; matrix = 288×384; slice number = 224; slice thickness = 0.6 mm; voxel size = 0.6×0.6×0.6 mm^3^, acquisition time = 7 minutes and 34 seconds. The injection of a single-dose (0.1 mmol per kilogram of body weight) gadolinium-based contrast agent (Magnevist; Schering, Berlin, Germany) was performed 5 minutes before the post-contrast scan with identical scanning parameters.

### DSA Acquisition

DSA was performed with a 5F catheter via the femoral artery to image the cervical and intracranial arteries. Both lateral and anterior-posterior projections were obtained from the double C arms of an Artis Zee biplane angiography system (Siemens, Munich, Germany). At each location, 7-8 mL of iodinated contrast medium (Jiejing Pharmaceutical, Shandong, China) was injected at a flow rate of 5 mL/sec. DSA was performed with a 40-cm field of view, a 1000 × 1000 matrix, and 0.4 × 0.4 mm^2^ spatial resolution.

### VWMRI Image Analysis

Before VWMRI image interpretation, one author generated postprocessed images of the craniocervical arteries, including multiplanar reformation, curved multiplanar reformation, and minimum intensity projection images, to visualize better the craniocervical lesions. Meanwhile, the image quality was evaluated by a reader with more than three years of experience on VWMRI. The image quality was assessed as follows: grade 1, excellent for diagnosis, high signal-to-noise ratio without artifacts, well-defined vessel wall and lumen borderline; grade 2, appropriate for diagnosis, average signal-to-noise ratio, only minor flow artifacts and distinguishable wall structures, but partially obscured vessel wall boundaries and lumen; and grade 3, inadequate for analysis, low signal-to-noise ratio and unidentifiable vessel boundaries, and major flow artifacts.

Two independent neuroradiologists with more than ten years of experience, blinded to clinical and DSA data, separately reviewed the 3D T1-SPACE images. The definition of imaging findings associated with CCAD diagnosis on 3D T1-SPACE images, including the IMH, patent double lumen, and intimal flap, was consistent with the prior study of Wu et al. [6]. IMH was defined as a crescent-shaped thickening of the vessel wall that was isointense/hyperintense on precontrast images. The patent double lumen was defined as a true and a false lumen with blood flow. An intimal flap was counted as a tear at some point in the artery's lining. For diagnostic test evaluation, referring to the recently provided criteria [1], 3D-VWMRI readers were required to express their diagnostic confidence for the presence of CCAD. The diagnosis of CCAD was interpreted according to a five-point confidence scale [16]: 1 (definitely absent), 2 (probably absent), 3 (uncertain), 4 (possibly present), and 5 (certainly present). In addition, 3D-VWMRI readers determined the presence/absence of intraluminal thrombus. The intraluminal thrombus was defined as luminal hyperintense filling on pre-contrast 3D T1-SPACE images, as well as an area of intraluminal contrast enhancement on post-contrast 3D T1-SPACE images [6]. In cases of disagreement on the identification of dissected location, the diagnostic confidence of dissection, and the presence of intraluminal thrombus, a third senior reader was invited to reach a consensus. All source images were assessed using commercial software packages (Osirix MD; Pixmeo SARL, Switzerland and Vessel Analysis; Surway Star Technology, China).

### DSA Image Evaluation

DSA images were evaluated with consensus decisions by two neurologists using the same software described above, both of whom were blinded to clinical and MRI information. An intraluminal thrombus on DSA images was defined as a local filling defect or a nonuniform opacification with irregular borders [17].

The DSA findings included the “pearl and string sign”, “string sign”, intimal flap, luminal dilation, tapered stenosis/occlusion, and irregular stenosis. The imaging signs associated with CCAD diagnosis on DSA included intimal flap, patent double lumen, “pearl and string sign”, “string sign”, and tapered stenosis or occlusion [18]. The “pearl and string sign” is defined as a fusiform aneurysmal dilatation with stenosis at proximal and/or distal sites. The “string sign” feature refers to a thin string of intravascular contrast material distal to a stenotic [19]. Irregular stenosis is stenosis that cannot be regarded as a “pearl and string sign”, “string sign”, or tapered stenosis.

**Table 1:** Participant characteristics.

Characteristics	All Patients (n=104)
Age (y)^*^	44.2 ± 13.2
Male patients	76 (73.1)
Body mass index, kg/m^2^^*^	25.2 ± 3.4
Smoker (current and former)	35 (33.7)
Alcohol, ≥ 3 drinks per week	23 (22.1)
Hypertension	40 (38.5)
Diabetes	10 (9.6)
Major risk factors	
Hyperlipidemia	31 (29.8)
Hyperhomocysteinemia	15 (14.4)
History of trauma	9 (8.7)
Major symptom and sign	
Headache and/or Neck pain	55 (52.9)
Dizziness	45 (43.3)
Horner's syndrome	4 (3.8)
Cranial nerve palsy	22 (21.2)
Acute cerebrovascular events	
Subarachnoid hemorrhage	9 (8.7)
Ischemic stroke	66 (63.5)
Transient ischemic attack	10 (9.6)
Onset prior to VWMRI, days^†^	9.5 (4.3-20.0)

Note: Applicable data are numbers of patients with percentages in parentheses.

* Mean values ± standard deviation.

† Data are medians, with interquartile range in parentheses.

### Reference Standard

Since no single reference standard for the diagnosis of CCAD was available, the final diagnosis results based on the clinical information, image data sets, and the results of follow-up were chosen to be the reference standard in the current study [20-22]. The final diagnosis of CCAD is a consensus of the attending neuroradiologists, neurosurgeries, and neurologists. The performance of 3D-VWMRI with a 3D T1-SPACE sequence in the detection of intraluminal thrombus using DSA served as the reference standard [23].

**Figure 1.**
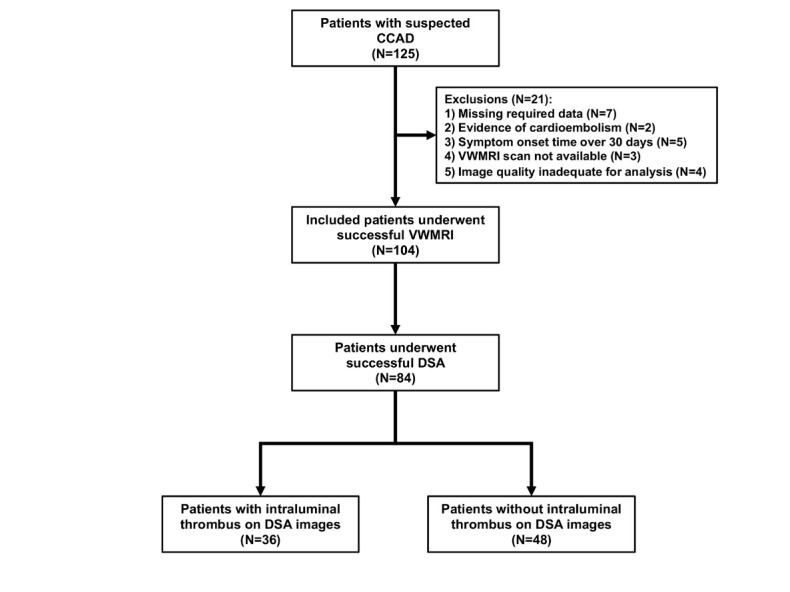
Study flowchart. 3D-VWMRI: three-dimensional vessel wall MRI, DSA: digital subtraction angiography.

### Statistical Analysis

Normal continuous variables were expressed as the mean ± standard deviation, whereas nonnormal data were expressed as the median and interquartile range. Categorical variables are described as counts (percentages). Comparisons between groups were conducted using the *t* test (normal data) or the Mann-Whitney *U* test (nonparametric data) for continuous variables and the chi-squared test for categorical variables.

CCAD diagnostic performance for 3D T1-SPACE compared to the final diagnosis was evaluated using receiver operating characteristic (ROC) analysis. The area under the ROC curve (AUC) value for each ROC analysis was reported. The 95% confidence interval (95% CI) was calculated for the AUC and the remaining applicable parameters. Sensitivity, specificity, positive predictive value (PPV), negative predictive value (NPV), and accuracy were calculated based on the local maximus corresponding to the cutoff values.

Sensitivity, specificity, PPV, NPV, and accuracy were used to assess the performance of the 3D T1-SPACE images in the detection of intraluminal thrombus using the DSA as the reference standard. Interobserver agreement was determined using the Cohen kappa coefficient for thrombus detection. The κ value was interpreted as follows: greater than 0.80, suggesting a high level of agreement, 0.61-0.80 substantial, 0.41-0.60 moderate, 0.21-0.40 fair, and 0-0.21 poor [24]. The diagnostic odds ratio (OR) was calculated to correlate the thrombus detected using the 3D T1-SPACE/DSA images and ischemic stroke. The statistical analysis was performed with R 3.6.1 (R Foundation for Statistical Computing, Vienna, Austria).

## RESULTS

### Patient Characteristics

Of the 125 patients, 104 patients (mean age, 44.2 years ± 13.2; 76 men) with successful 3D-VWMRI met the inclusion criteria and were enrolled in our study (Fig. 1). Twenty-one patients were excluded from the study because of missing conventional imaging data (7 patients), evidence of cardioembolism (2 patients), onset time of symptoms over 30 days (5 patients), unavailability of 3D-VWMRI (3 patients), and 3D-VWMRI image quality of grade 3 (4 patients). Eighty-four of the 104 patients (80.8%) successfully underwent the DSA test (median duration between 3D-VWMRI and DSA: 3.0 days; interquartile range: 2.0 to 4.8 days). The clinical characteristics of the 104 included patients are summarized in Table 1.

**Figure 2.**
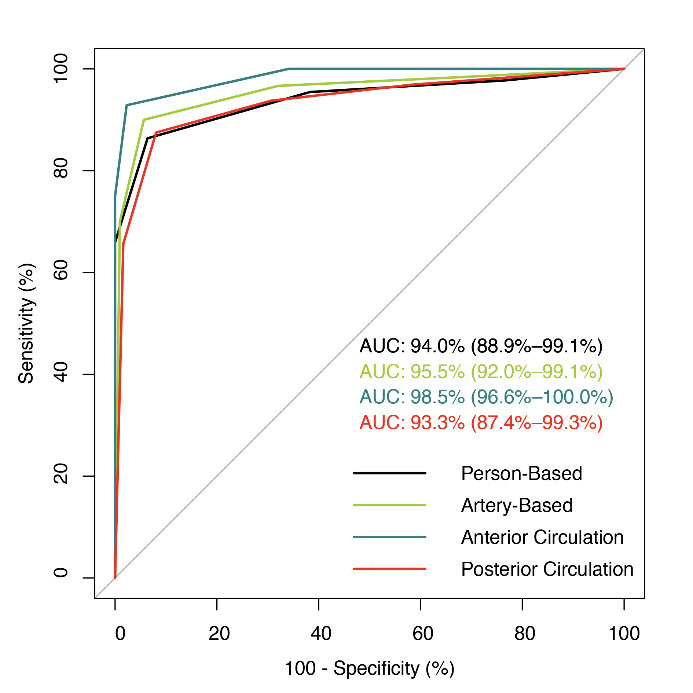
ROC curve analysis for detecting CCAD for all image sets in suspicious patients, suspected arteries, suspected arteries of the anterior circulation, and suspected arteries of the posterior circulation. The ROC curves show the AUC values with their 95% confidence intervals in parentheses. CCAD: craniocervical artery dissection, AUC: area under the receiver operating characteristic curve.

### Image Quality

The 3D T1-SPACE images from 16 of the 104 recruited patients (15.4%) were classified as grade 2, which was due to only minor impacts from relevant artifacts. The 3D T1-SPACE image sets from the remaining 88 patients (84.6%) were classified as grade 1.

### Diagnostic Performance of 3D-VWMRI for CCAD

**Figure 3.**
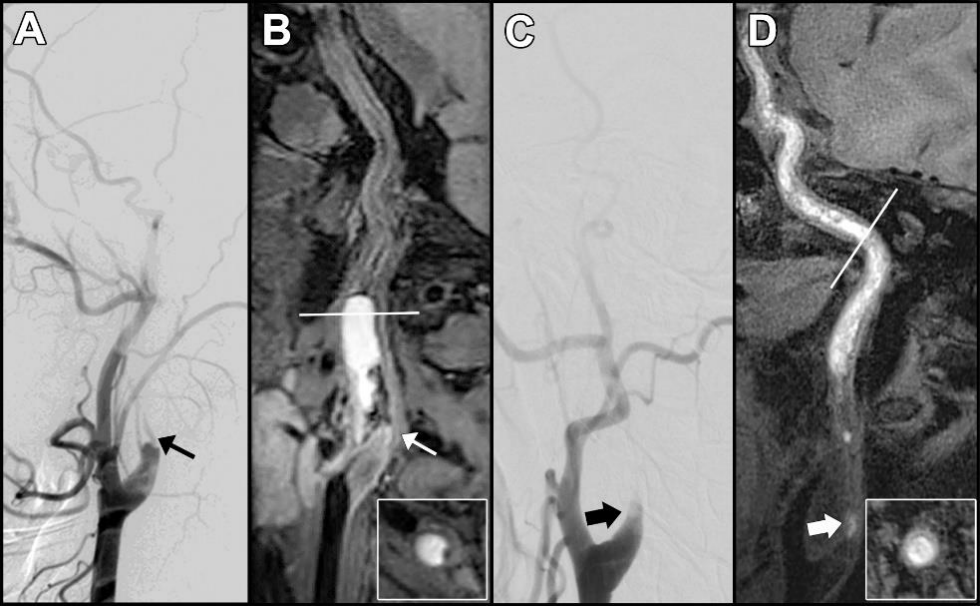
Representative cases of cutoff and tapered occlusions in internal carotid arteries. (A) The case of cutoff occlusion on DSA images. A thin black arrow indicates the cutoff occlusive lesion. (B) The corresponding case of cutoff occlusion on the 3D T1-SPACE images. A thin white arrow displays the cutoff occlusive lesion. The curved planar reformation of 3D T1-SPACE axial images (B, white-edged rectangles) presents the IMH in the cervical segment of the left internal carotid artery, representing a typical sign of cervical artery dissection. (C) The case of tapered occlusion on DSA image. A wide black arrow indicates a tapered occluded lesion. (D) A noncontrast 3D T1-SPACE image shows a long hyperintense filling in the right internal carotid artery from the cervical segment extending into the ophthalmic segment. A wide white arrow indicates the occlusion site. IMH: intramural hematoma.

The final diagnosis was made for 91 patients with 166 arteries with suspected dissection lesions. Seventy-two (43.4%) of the 166 arteries were located in the anterior circulation, and 94 (56.6%) were located in the posterior circulation. Supplementary Table 1 summarizes the VWMRI findings of CCAD. The confidence score of forty-three arteries was rated as 5 points. Seventeen arteries had a score of 4 on the confidence scale. One hundred and six arteries were scored as 3 points (32 patients), 2 points (38 patients), and 1 point (36 patients), respectively. The 3D T1-SPACE sequences offered diagnostic performance for both per-patient and per-artery analyses with AUC values of 0.94 (95% CI: 0.90-0.99) and 0.96 (95% CI: 0.92-0.99), respectively (Fig. 2). For the per-patient and per-artery assessments, the 3D T1-SPACE images showed high accuracy (90.1% and 92.8%, respectively) in the diagnosis of dissection compared to the final diagnosis (Table 2). The DSA findings of 95 arteries with both successful 3D T1-SPACE and DSA images are summarized in Supplementary Table 2. Two represented cases with different types of occlusion on DSA images are shown in Figure 3.

### Thrombus Detection with 3D-VWMRI Using DSA as the Reference

**Figure 4.**
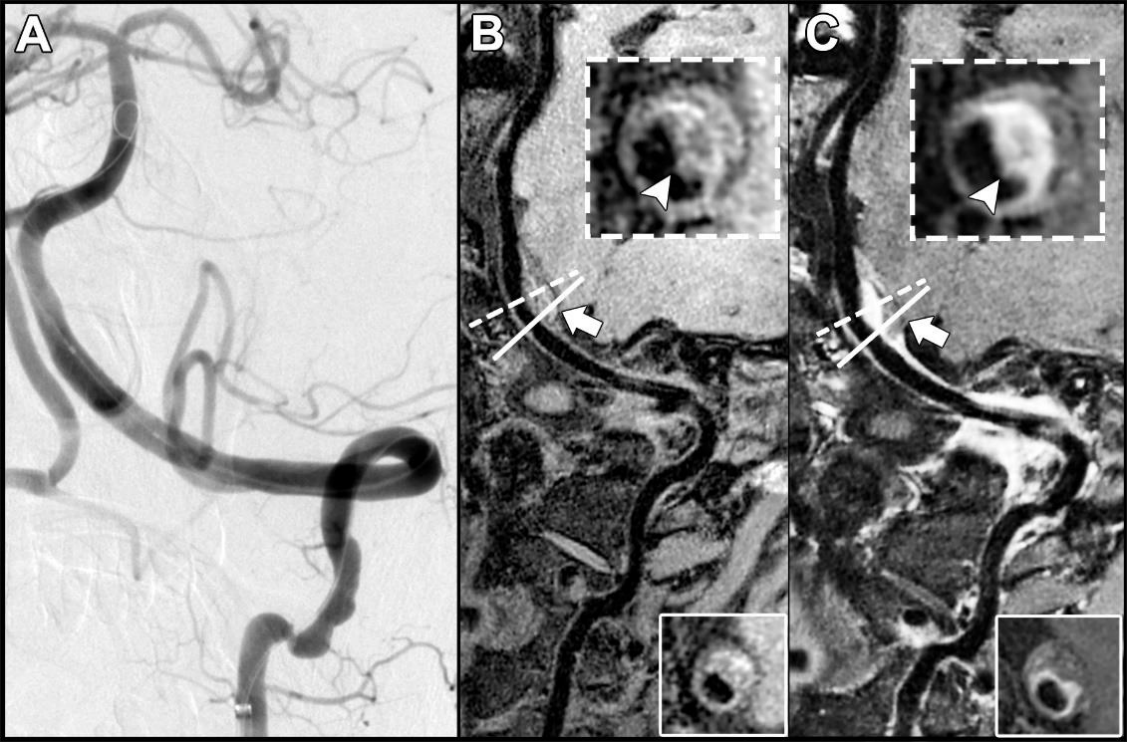
A representative case with an intraluminal thrombus presented on 3D T1-SPACE images but absent on DSA images. (A) A DSA image shows a normal V4 segment in the left vertebral artery. (B and C) Pre- and postcontrast 3D T1-SPACE images demonstrate a dissected lesion with IMH (indicated by solid box and white arrows) on the vessel wall of the V4 segment of the vertebral artery. A 3D T1-SPACE sequence was found to be useful to distinguish a mural thrombus from IMH of the left vertebral artery (indicated by dotted box and white arrowheads). IMH: intramural hematoma.

Among the 95 arteries of 84 patients with both successful 3D T1-SPACE and DSA tests, 39 arteries of 36 patients had intraluminal thrombus on DSA images. The two 3D-VWMRI readers each identified 38 and 36 of these arteries, yielding sensitivities of 97.4% and 92.3%, respectively. The interobserver agreement between the two 3D-VWMRI readers for thrombus detection was high (κ = 0.89, 95% CI: 0.80-0.98, *P* < .001). Fifty-seven arteries were detected with intraluminal thrombus using 3D T1-SPACE sequence with consensus, resulting in a sensitivity of 97.4%, a specificity of 66.1%, and an accuracy of 79.0% for thrombus detection with the DSA used as the reference standard (Table 3). Figure 4 displays a case with an intraluminal thrombus present on 3D T1-SPACE images but absent on DSA images.

**Table 2:** Diagnostic performance of 3D T1-SPACE for CCAD compared to final diagnosis.

Variable	Person-based	Artery-based	Anterior Circulation	Posterior Circulation
Sensitivity (%)	86.4 (72.7, 94.8)	90.0 (79.5, 96.2)	92.9 (76.5, 99.1)	87.5 (71.0, 96.5)
	[38/44]	[54/60]	[26/28]	[28/32]
Specificity (%)	93.6 (82.5, 98.7)	94.3 (88.1, 97.9)	97.7 (88.0, 99.9)	91.9 (82.2, 97.3)
	[44/47]	[100/106]	[43/44]	[57/62]
PPV (%)	92.7 (80.1, 98.5)	90.0 (79.5, 96.2)	96.3 (81.0, 99.9)	84.8 (68.1, 94.9)
	[38/41]	[54/60]	[26/27]	[28/33]
NPV (%)	88.0 (75.7, 95.5)	94.3 (88.1, 97.9)	95.6 (84.9, 99.5)	93.4 (84.1, 98.2)
	[44/50]	[100/106]	[43/45]	[57/61]
Accuracy (%)	90.1 (82.1, 95.4)	92.8 (87.7, 96.2)	95.8 (88.3, 99.1)	90.4 (82.6, 95.5)
	[82/91]	[154/166]	[69/72]	[85/94]

Note: The numbers in parentheses are 95% CIs, and numbers in brackets are raw data. PPV: positive predictive value, NPV: negative predictive value.

### The Presence/Absence of a Thrombus on 3D-VWMRI or DSA and Ischemic Stroke

Nine of 84 patients with both successful 3D T1-SPACE and DSA tests had subarachnoid hemorrhages, and they were not included in the following analysis considering the difference between hemorrhagic stroke and ischemic stroke in etiology and treatment. Among the 86 vessels of the remaining 75 patients, 65 (76%) vessels were not occluded. Fifty-one vessels of 49 patients had ischemic stroke in the corresponding vessel territory.

**Figure 5.**
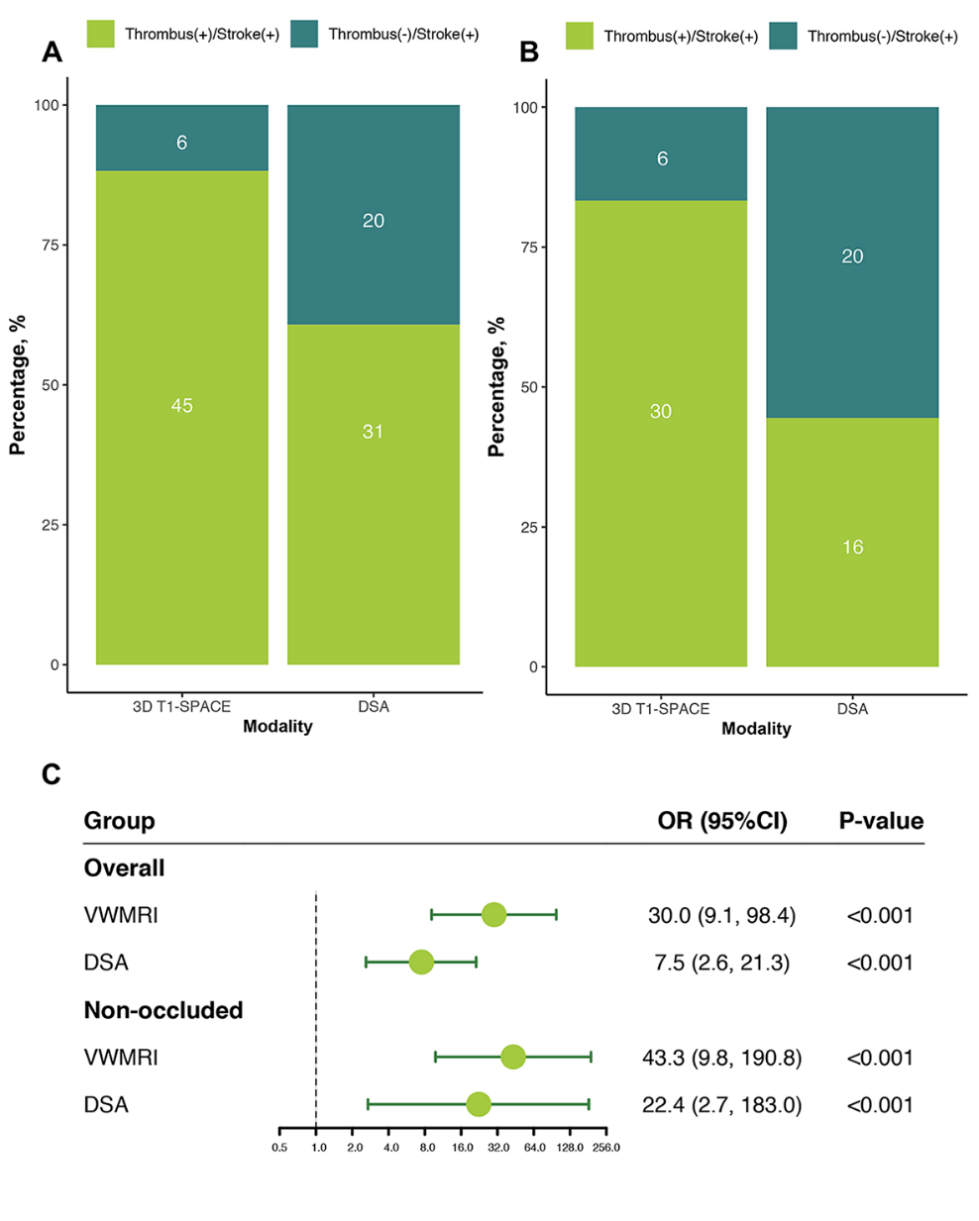
The presence/absence of thrombus on 3D T1-SPACE or DSA images and ischemic stroke. In the overall analysis of 86 arteries (A) and analysis of 65 nonoccluded arteries (B), intraluminal thrombus-positive cases on 3D T1-SPACE images were more prevalent in the territory ischemic stroke group than in DSA images. The numbers in stacks are the counts of arteries. (C) Diagnostic odds ratio (OR) and 95% CIs of intraluminal thrombus detected using 3D-VWMRI or DSA for territorial ischemic stroke.

In the overall analysis, the intraluminal thrombus presented on 3D T1-SPACE images was more prevalent in the group of territorial ischemic strokes than that presented on DSA (88.2% vs. 60.8%) (Fig. 5A). The intraluminal thrombus identified using 3D T1-SPACE was strongly associated with ischemic stroke (OR: 30.0, 95% CI: 9.1-98.4, *P* < 0.001) .

**Figure 6.**
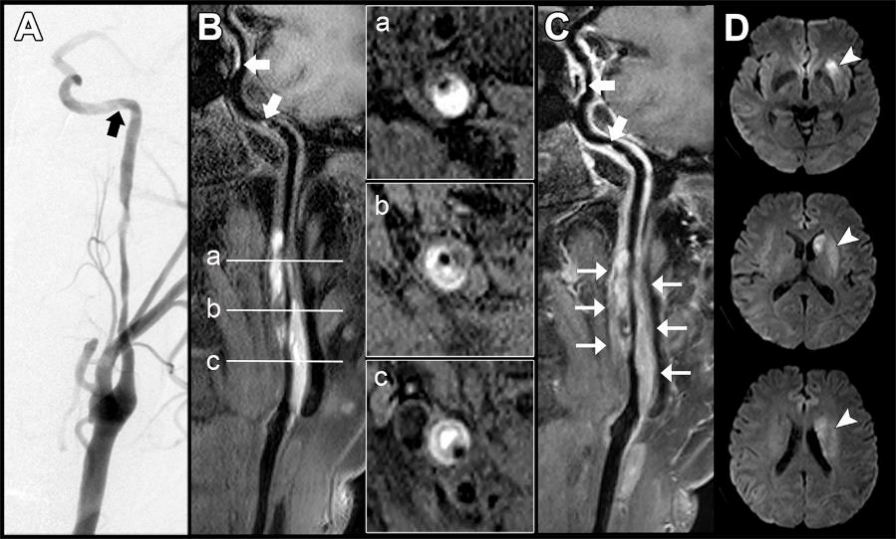
A case with left cervical artery dissection and territorial ischemic stroke. (A) The unilateral DSA image of this patient shows irregular stenosis and a triangular filling defect (black arrow) in the left internal carotid artery. (B) Curved planar reformation of noncontrast 3D T1-SPACE with axial images (white-edged rectangles of planes a, b, and c). (C) The contrast 3D T1-SPACE image is indicative of intramural hematoma (thin white arrows) and intraluminal thrombus (white arrows). (D) Diffusion-weighted imaging presents infarction of the left basal ganglia and periventricular white matter (white arrowheads).

In the analysis of nonoccluded vessels, 3D T1-SPACE found 14 more vessels with intraluminal thrombi than DSA among 36 vessels with territorial ischemic stroke (Fig. 5B). Among 29 vessels without territorial ischemic stroke, two more vessels were detected with intraluminal thrombi using 3D T1-SPACE than using DSA. The ORs of intraluminal thrombus presented on 3D T1-SPACE or DSA in nonocclusive vessels for territorial ischemic stroke were 43.3 (95% CI: 9.8-190.8, *P* < .001) and 22.4 (95% CI: 2.7-183.0, *P* < .001), respectively (Fig. 5C). Representative images are shown in Figure 6.

**Table 3:** Thrombus detection with 3D T1-SPACE in 95 arteries using DSA as reference.

Variable	VWMRI (Consensus)	VWMRI Reader1	VWMRI Reader2
Sensitivity (%)	97.4 (86.5, 99.9)	97.4 (86.5, 99.9)	92.3 (79.1, 98.4)
	[38/39]	[38/39]	[36/39]
Specificity (%)	66.1 (52.2, 78.2)	71.4 (57.8, 82.7)	69.6 (55.9, 81.2)
	[37/56]	[40/56]	[39/56]
PPV (%)	66.7 (58.0, 74.3)	70.4 (61.0, 78.3)	67.9 (58.5, 76.1)
	[38/57]	[38/54]	[36/53]
NPV (%)	97.4 (84.1, 99.6)	97.6 (85.2, 99.6)	92.9 (81.2, 97.5)
	[37/38]	[40/41]	[39/42]
Accuracy (%)	79.0 (69.4, 86.6)	82.1 (74.9, 89.2)	79.0 (69.4, 86.6)
	[75/95]	[78/95]	[75/95]

## DISCUSSION

Our investigation of 3D T1-SPACE sequences at 3.0-T MR system in patients suspected of CCAD yielded three major findings. First, 3D T1-SPACE was found to have high diagnostic performance for CCAD at both the patient and artery levels. Second, 3D T1-SPACE demonstrated high sensitivity for detecting intraluminal thrombus when compared to DSA. Third, 3D T1-SPACE had added value in the detection of intraluminal thrombus associated with territorial ischemic stroke, especially in the identification of nonocclusive intraluminal thrombus.

Imaging techniques have been widely used for detecting CCAD. Instead of directly imaging dissected lesions, most of the conventional modalities demonstrate vascular abnormalities depending on altered luminal morphology, failing to entirely characterize lesions that reside within the vessel wall [25]. The drawback of lumenography was particularly evident when attempting to assess occluded arteries due to the invisibility of the distal segments from occlusive sites, potentially leading to false-negative or false-positive results for dissection diagnosis. Additionally, there are many underlying pathologic processes in the distal segments of occluded vessels. In terms of CCAD, the intraluminal growth of IMH in the dissected artery narrows the vascular lumen, thereby possibly resulting in occlusion or promoting the propagation of thrombus in the affected artery. Hemodynamic infarction or secondary embolic infarction into distal arteries may consequently occur [26]. Luminal angiography fails to provide the above information. Several examples from the present study illustrate these established limitations. Some imaging methods, such as Doppler ultrasonography and CTA, can depict the vessel lumen and wall [27, 28]. However, they have limited utility in evaluating basicranial or transcranial segments, which is due to the influence of bone and low-contrast presentation.

3D-VWMRI with comprehensive head and neck coverage can reveal the vessel wall and intravascular changes of craniocervical arteries in a single acquisition with good dark blood contrast [7]. Unlike luminal imaging techniques, 3D-VWMRI is capable of directly targeting specific CCAD image features, including the IMH, intimal flap, and double lumen [29], and thus can improve the diagnostic confidence of CCAD [21], which is particularly important in CCAD cases wherein conventional imaging displays nonspecific features such as tapering stenosis and flame signs. 3D-VWMRI with volumetric isotropic turbo spin-echo acquisition (VISTA; Philips Healthcare, Best, Netherlands) sequence had a sensitivity of 95.2% and specificity of 100% for the diagnosis of cervical artery dissection [20]. Using a 3D T1-SPACE sequence with complete neck and head coverage, we further demonstrated the excellent performance of 3D-VWMRI for CCAD diagnosis. Apart from the diagnosis of CCAD, a recent study illustrated that 3D-VWMRI offers additional information, including thrombosis and the full collapse of occluded vessels [30], which is helpful to investigate the cause of stenosis or occlusion and the mechanism of the ischemic event.

Thromboembolism is the essential ischemic stroke mechanism in CCAD. The intimal flap and irregular surface of the vessel wall may reflect endothelial damage, which activates thrombus formation and may ultimately lead to occlusion or embolism [31]. Ischemic stroke in patients with CCAD is frequently associated with signs of thrombotic mechanisms observed upon imaging [6, 32]. Coppenrath et al. [33] demonstrated that intraluminal contrast enhancement on 2D-T1-weighted images revealed intraluminal thrombus formation and was strongly correlated with territorial ischemic stroke with an odds ratio of 32.0 (95% CI: 3.6-281) in 33 patients with spontaneous cervical artery dissection. A study by Jang et al. [34] evaluated the utility of appending 3D-VISTA to the stroke MRI protocol, including diffusion-weighted imaging and/or susceptibility-weighted imaging for detecting intracranial arterial thrombus in patients with stroke. The results demonstrated that the inclusion of 3D-VISTA in the study’s stroke MRI protocol improved the detection of occlusive intraluminal thrombi in anterior and middle cerebral arteries with higher accuracy than using the stroke MRI protocol. Our study found that 3D T1-SPACE provides a panoramic view of dissected vessels and a whole picture of the local and distal thrombosis burden in culprit arteries at a one-step examination. Furthermore, we investigated the added value of 3D T1-SPACE to detect nonocclusive intraluminal thrombus related to territorial ischemic stroke.

Our comparative work found that 97.4% of intraluminal thrombi presented on DSA images could be detected using 3D T1-SPACE in artery-by-artery analysis. Furthermore, 3D-VWMRI readers observed intraluminal thrombus located on 88.2% of culprit arteries in the ischemic stroke group, but 33.1% of vessels therein were missed on DSA images. 3D T1-SPACE can more directly indicate the existence of intraluminal thrombi and more frequently found multiple intraluminal thrombi in culprit arteries than DSA. Compared to that in occluded vessels, ischemic stroke in the corresponding territory of the nonocclusive vessel is more likely to be caused by artery-to-artery embolization. The 3D T1-SPACE also exhibited good detective efficacy of intraluminal thrombus associated with ischemic stroke in nonoccluded vessels during our study. These findings implied that 3D T1-SPACE could be a valuable tool for early and noninvasive evaluation/prediction of the risk of ischemic stroke caused by thromboembolism and thus should be practical for planning future therapeutic strategies in patients with suspected or predetermined CCAD.

However, we also found it was difficult to distinguish intraluminal thrombus from subacute IMH in occlusive arteries with hyperintense filling by 3D-VWMRI alone. Follow-up examinations in these cases may be necessary to arrive at an accurate identification. Additionally, an important pitfall of 3D-VWMRI is that flow-related enhancement often mimics the intimal flaps, thrombus, and even IMH. Flow-related artifacts more frequently appear in regions with low blood flow rates and basicranial segments [7], potentially leading to false-positive results or diagnostic uncertainty when performing the analysis. Minimizing the impact of slow blood flow and the fat signal can be essential to obtain high-quality 3D-VWMRI images, particularly for vertebral arteries requiring high tissue contrast.

The current study has some limitations. First, this study was limited to a single center for patient recruitment and image evaluation. Second, all of the information for each patient, including the 3D-VWMRI images, was used to define the reference standard for diagnosis, which may lead to review bias of the sensitivity and specificity assessments. Third, twenty patients were excluded from the comparative analysis of thrombus detection because of a lack of DSA examination. In addition, 3D-VWMRI may be unacceptable to patients who cannot withstand long scan durations. These limitations may have resulted in a selective bias for our study. Fourth, in the present study, we did not perform pathology or age differentiation on the intraluminal thrombus. An acute thrombus appears strongly hypointense, and a subacute thrombus appears hyperintense on T2-weighted images [35], which may help to better define the age of the thrombus. We did not perform T2-weighted imaging due to scan time constraints. Additional multicenter, longitudinal studies are needed to assess these promising 3D-VWMRI methods further and to determine whether patients with intraluminal thrombus signals on 3D-VWMRI images are at greater risk for a worse clinical outcome or recurrent stroke regarding CCAD. Whether patients with thrombosis could profit from more aggressive antithrombotic treatment also remains to be investigated using 3D-VWMRI in future studies.

In conclusion, a 3D T1-SPACE sequence with a 3.0-T MR system was a viable option for the diagnosis of CCAD. In addition, 3D T1-SPACE can provide a direct and accurate depiction of intraluminal thrombus that is strongly related to territorial ischemic stroke compared with DSA, which is an added value of VWMRI. The latter finding indicates that noninvasive 3D T1-SPACE holds promise to better triage patients with impending acute ischemic stroke in clinical practices associated with CCAD.

## Supplementary Materials

The Supplemenantry data can be found online at: www.aginganddisease.org/EN/10.14336/AD.2021.0502.


