# Supplementary material for: The Added Value of Vessel Wall MRI in the Detection of Intraluminal Thrombus in Patients Suspected of Craniocervical Artery Dissection
Source: Aging Dis. 2021 Dec 1;12(8):2140–50. doi: 10.14336/AD.2021.0502 (PMC8612619; doi:10.14336/AD.2021.0502)
Supplement: Supplementary file 1 [file AD-12-8-2140-s.pdf]

SUPPLEMENTARY DATA

The Added Value of Vessel Wall MRI in the Detection of Intraluminal Thrombus in Patients Suspected of Craniocervical Artery Dissection

Yuehong Liu[1,2,#], Sijie Li[3,#], Ye Wu[2], Fang Wu[2], Ying Chang[4], Haibin Li[5], Xiuqin Jia[1], Luca Saba[6], Xunming Ji[3], Qi Yang[1,7,8*], on behalf of the WISP Investigators

© 2021. Liu Y et al. Published online at http://www.aginganddisease.org/EN/10.14336/AD.2021.0502

SUPPLEMENTARY DATA

Supplementary Table 1. The 3D T1-SPACE Findings for 166 Suspicious Arteries with Final Diagnosis.

**3D T1-SPACE Findings**	**No. of Arteries (n=166)**
Specific Findings	61 (36.7%)
IMH	47 (28.3%)
Intimal Flap	11 (6.6%)
Patent Double Lumen	3 (1.8%)
Non-specific Findings	105 (63.3%)

Note: Applicable data are numbers of arteries with percentages in parentheses. The imaging features without any of the specific findings, including intramural hematoma (IMH), intimal flap, and double lumen are non-specific findings.

Supplementary Table 2. The DSA Findings for 95 Arteries with Successful 3D T1-SPACE and DSA.

DSA Findings	No. of Arteries (n=95)
Pearl and String Sign	9 (10%)
String Sign	2 (2%)
Intimal Flap	1 (1%)
Intimal Flap and Dilation	1 (1%)
Dilation	21 (22%)
Irregular Stenosis	35 (37%)
Tapered Occlusion	7 (7%)
Non-tapered Occlusion	15 (16%)
Normal	4 (4%)
Intraluminal Thrombus (+)	39 (41%)

Note: Applicable data are numbers of arteries with percentages in parentheses.

© 2021. Liu Y et al. Published online at http://www.aginganddisease.org/EN/10.14336/AD.2021.0502
